# Supplementary figures and images for: Proteomics data in vitiligo: a scoping review
Source: Front Immunol. 2024 Apr 23;15:1387011. doi: 10.3389/fimmu.2024.1387011 (PMC11074361; doi:10.3389/fimmu.2024.1387011)

# Supplementary Material

Supplementary Figure 1. PRISMA Flow chart

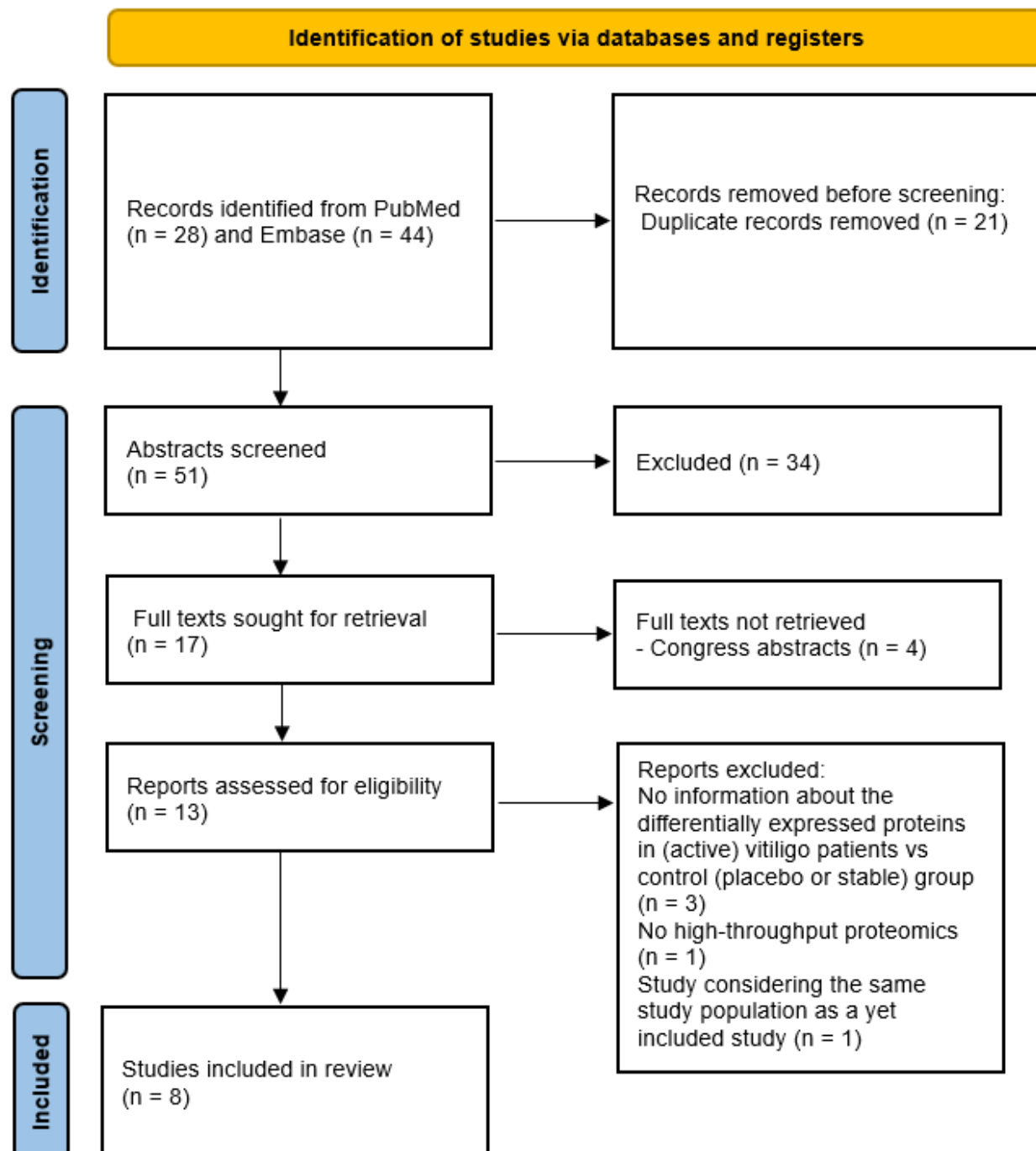

Supplement: Supplementary file 1 [file DataSheet_1.pdf]
